# Lattice modulation strategies for 2D material assisted epitaxial growth

**DOI:** 10.1186/s40580-023-00388-0

**Published:** 2023-08-25

**Authors:** Qi Chen, Kailai Yang, Meng Liang, Junjie Kang, Xiaoyan Yi, Junxi Wang, Jinmin Li, Zhiqiang Liu

**Affiliations:** 1 Research and Development Center for Semiconductor Lighting Technology, Institute of Semiconductors, Chinese Academy of Sciences, Beijing, 100083 China; 2 Center of Materials Science and Optoelectronics Engineering, University of Chinese Academy of Sciences, Beijing, 100049 China

**Keywords:** Remote epitaxy, vdW epitaxy, 2D material

## Abstract

As an emerging single crystals growth technique, the 2D-material-assisted epitaxy shows excellent advantages in flexible and transferable structure fabrication, dissimilar materials integration, and matter assembly, which offers opportunities for novel optoelectronics and electronics development and opens a pathway for the next-generation integrated system fabrication. Studying and understanding the lattice modulation mechanism in 2D-material-assisted epitaxy could greatly benefit its practical application and further development. In this review, we overview the tremendous experimental and theoretical findings in varied 2D-material-assisted epitaxy. The lattice guidance mechanism and corresponding epitaxial relationship construction strategy in remote epitaxy, van der Waals epitaxy, and quasi van der Waals epitaxy are discussed, respectively. Besides, the possible application scenarios and future development directions of 2D-material-assisted epitaxy are also given. We believe the discussions and perspectives exhibited here could help to provide insight into the essence of the 2D-material-assisted epitaxy and motivate novel structure design and offer solutions to heterogeneous integration via the 2D-material-assisted epitaxy method.

**Graphical Abstract:**

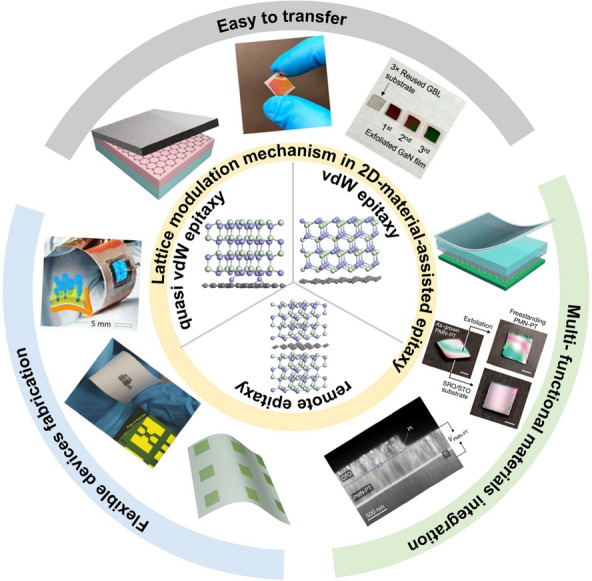

## Introduction

Epitaxial growth, a technique to fabricate single-crystalline membranes, has experienced a revolution from the homo- to the hetero-one. In this process, though still governed by the covalent interaction, the application of strain engineering, such as introducing a low-temperature buffer layer or superlattice structure, widens the substrate options to some extent [1]. This solves the lack of native substrate problem and brings advantages such as cost reduction and yield increase, moreover, offers more possibilities for structure and device design. However, the lattice and thermal mismatch have been a long-exist problem in heteroepitaxy. For the small lattice mismatch (usually less than 3%) scenarios, a pseudomorphic growth is first expected to happen, during which the epilayer would endure the lattice distortion and strain accumulation to maintain the crystalline quality. Once reaching the critical thickness, the epilayer tends to recover its own lattice constant and release the stored strain by the formation of dislocations. For the large lattice mismatch ones, misfit dislocations would immediately occur at the heterointerface to accommodate the large mismatch-induced strain. As a result, in principle, the crystalline quality of the hetero-epilayer is not as satisfied as that of the homoepitaxial ones. Besides, the limitation of lattice symmetry also decides that it is impossible to implement heteroepitaxy on arbitrary substrates, which greatly limits the integration of multi-functional materials via the direct epitaxy method.

A layered material growth method proposed in 1984, namely, the van der Waals (vdW) epitaxy, gives inspiration for the further development direction of the bulk material epitaxy [2]. Utilizing NbSe_2_/MoS_2_ system as an example, Koma et al. demonstrate the possibility of materials growth on a dangling-bonds-free surface guided by the vdW force. In this scenario, the epilayer is not constrained to the substrate by covalent interaction, thus the epilayer tends to preserve its own lattice structure and not establish the epitaxial relationship with the substrate as that happens in the traditional covalent epitaxy. This growth paradigm offers hope for fully breaking the substrate restriction and is favorable for implementing dissimilar material integration. However, due to the unavoidable presence of dangling bonds on the bulk materials surface, the vdW epitaxy strategy could not be straightly replicated to bulk crystals growth.

The discovery of layered 2D materials as well as their superior and abundant properties gives a chance to enrich the conventional material set and the fabrication method [3–6]. Ever since the first 2D material graphene is successfully fabricated through the mechanical exfoliation method from graphite, efforts have been made to explore new members of 2D materials and the synthesis method [7–11]. With years of development, wafer-size 2D materials, including graphene, h-BN, and transition metal dichalcogenides (TMDCs), could be fabricated via the chemical vapor deposition method [12–17]. Furthermore, new materials and devices are developing rapidly. Park et al. implement the VSe_2_ growth on the epitaxial graphene-covered SiC substrate. The as-obtained heterostructure exhibit ultrafast and efficient interlayer hot electron transfer characteristics and interlayer coupling effects, which shows the potential of being the layered hot electron injecting material [18]. By taking advantage of emerging technologies, such as machine learning analysis, one could better understand the growth dynamics and control the layer thicknesses of the ultrathin 2D materials, which could greatly benefit the fabrication of 2D/2D heterostructures with desired performance [19]. Besides, utilizing the transferable characteristic of the 2D materials, one could create a dangling-bonds-free growth front on arbitrary substrates and then it is possible for bulk materials to fulfill “vdW” epitaxy, or generally speaking, the 2D-material-assisted epitaxy, on a 2D/3D complex substrate. Many studies have demonstrated single crystal growth via 2D-material-assisted epitaxy and find that as-grown membranes show great advantages in transferable, strain relaxation, and dislocation density reduction, compared with their counterparts obtained via covalent epitaxy [20–30]. Though these experimental results are fascinating, the theoretical framework of 2D-material-assisted epitaxy has not been elucidated straightforwardly and completely. The dangling-bonds-free surface brings opportunities to relieve the long-existed lattice mismatch problem, while also leaving some confusion on the lattice modulation mechanism. Generally speaking, the proposed lattice modulation strategies in 2D-material-assisted epitaxy could be categorized into three cases: (1) remote epitaxy, where the epilayer still inherits the substrate lattice regardless of the presence of the 2D material interlayer; (2) vdW epitaxy, where the epilayer is modulated by the 2D material and not affected by the underlying substrate; (3) quasi vdW epitaxy, where the artificial potential fluctuation created by the intentionally doped 2D material governs the epitaxial growth. Understanding the above-mentioned modulation mechanisms is essential for the successful implementation and proper application of 2D-material-assisted epitaxy.

In this work, we systematically review the lattice modulation mechanisms in 2D-material-assisted epitaxy and give feasible development and application directions of 2D-material-assisted epitaxy. Firstly, based on the concept of lattice transparency and the understanding of the potential relay effect, the lattice modulation mechanism and the possible application of remote epitaxy are discussed. Subsequently, we illustrate the impact of the lattice mismatch in the lattice modulating process in vdW epitaxy. Suggestions for utilizing vdW epitaxy to achieve material growth on arbitrary substrates are also provided. Finally, the possibilities of using quasi vdW epitaxy to manipulate the properties of the epitaxial structure are presented. The discussions and perspectives exhibited in this review may offer help to understand the essence of 2D-material-assisted epitaxy and motivate novel structure and device design via a 2D-material-assisted epitaxy method.

## Modulation mechanisms in 2D-material-assisted epitaxy

Date back in 2010, Chung et al. exhibit the possibility of nitrides growth on the 2D material [31]. Placing a mechanically exfoliated graphene as the buffer layer on a sapphire substrate, assisted with O_2_ plasma treatment and ZnO nano-walls growth procedure, a single-crystalline GaN epilayer is successfully obtained and provided a template for the subsequent light emitting diode (LED) structure growth. The as-fabricated LED shows visible blue emission at room temperature and could be transferred onto various target substrates via the mechanical lift-off method. This is regarded as an example of utilizing the optical characteristics of nitrides and mechanical properties of graphene, which demonstrates the potential of the 2D-material-assisted epitaxy for implementing heterogeneous integration [32]. Many other attempts have also been reported [23, 33–37] Kim et al. report nitrides growth on epitaxial graphene-covered SiC substrate in 2014 [38]. The steps of the epitaxial graphene could provide periodic nucleation sites for the subsequent GaN growth, which is beneficial for removing the extra interfacial buffer growth process and improving the transport characteristics at the heterointerface. With the careful control of growth kinetics, a single-crystalline GaN epilayer is obtained, the quality of which is comparable to that of growth via the AlN-buffer-assisted epitaxy strategy. Besides, the weak interaction at the heterointerface allows the epitaxial structure to be exfoliated easily. The as-exfoliated GaN film has an atomistic smooth surface, which permits the direct bonding of the released GaN onto the SiO_2_/Si (100) substrate and enables the reuse of the original SiC substrate. Hong et al. perform the InAs and GaAs nanostructures growth on graphic films in 2011 [39]. They illustrate that the growth behavior of 3D nano architectures on the 2D material surface is closely related to the lattice coherency at the 3D/2D heterointerface and the surface roughness of the 2D material. Compared with GaAs, it is found that the nearly lattice-matched InAs is more likely to form nanowires arrays on the graphitic surface. With a proper surface engineering strategy, density-controllable InAs nanowire arrays on the graphitic films could be achieved. This brings valid instructions for producing high-yield, uniform, and controllable nanostructures on the dangling-bond-free surface and promotes the development of various semiconductor/graphene hybrid junction electronics and optoelectronics. Shin et al. demonstrate ultrathin GaAs- and GaN-based LED structures on 2D material-covered substrates, respectively, and subsequently use the 2D-materials-based layer transfer technique to transfer and integrate the as-obtained LEDs for constructing a vertical-stacked full-color micro-LED array, which may further advance vertical micro display technology [40]. Assisted with graphene, transferable complex-oxide single-crystalline membranes are also successfully obtained and could be used for fabricating 3D/2D heterostructures, which is favorable for tailoring materials functionalities and studying the novel interface phenomena [41]. Using a nanopatterned graphene interlayer, Kim et al. achieve antiphase-boundaries-free compound semiconductor epitaxial growth on elemental semiconductor substrates, and the epilayers could be readily exfoliated with good controllability, which offers a new pathway for fabricating hetero-integrated multifunctional systems [42].

These mentioned-above results exhibit the possibility of 2D-material-assisted epitaxy in cutting the cost of the substrate and achieving dissimilar materials integration via a direct stacking strategy [43]. Along with the tremendous experimental demonstrations of 2D-material-assisted epitaxy, efforts are made to explore the modulation mechanisms behind it. Alaskar et al. investigate the adatoms adsorption and diffusion behaviors on the graphene surface in 2014 [44]. They find that with the inclusion of As or Ga pre-layer, the surface energy of graphene could be modified to promote the layer-by-layer growth mode. While the nucleation sites and density of III-V nucleus on graphene could be controlled by selecting the upcoming adatoms. This provides a primary guideline for the construction of the growth front on chemical inertness 2D material surfaces. Hong et al. study the vdW heterointerface of the InAs/suspended graphene/InAs system in 2013 [22]. The obtained binding energy and interaction gap offer a valuable criterion for judging the binding type of the heterointerface in 2D-material-assisted epitaxy.

With these experimental and theoretical findings, a platform is provided for the subsequent study of the 2D-material-assisted epitaxy. Benefiting from this, the theoretical framework of lattice modulation mechanisms in 2D-material-assisted epitaxy, including the remote epitaxy, vdW epitaxy, and quasi vdW epitaxy, develop rapidly.

### Remote epitaxy

Many studies have demonstrated bulk materials growth on 2D-material-covered single-crystalline substrates. The obtained epilayers have comparable crystalline quality to the heteroepitaxial ones and exhibit advantages in the exfoliation and transfer process, which greatly satisfies the flexible device fabrication and opens a pathway for heterogeneous integration through the direct stacking method [45–53]. However, after placing a 2D material buffer layer on a crystalline substrate, the surface dangling bonds seem to be eliminated, which results in long-term confusion about the nucleation and orientation modulation mechanism for the epitaxial growth on the 2D-material-covered single-crystalline substrate.

**Fig. 1:**
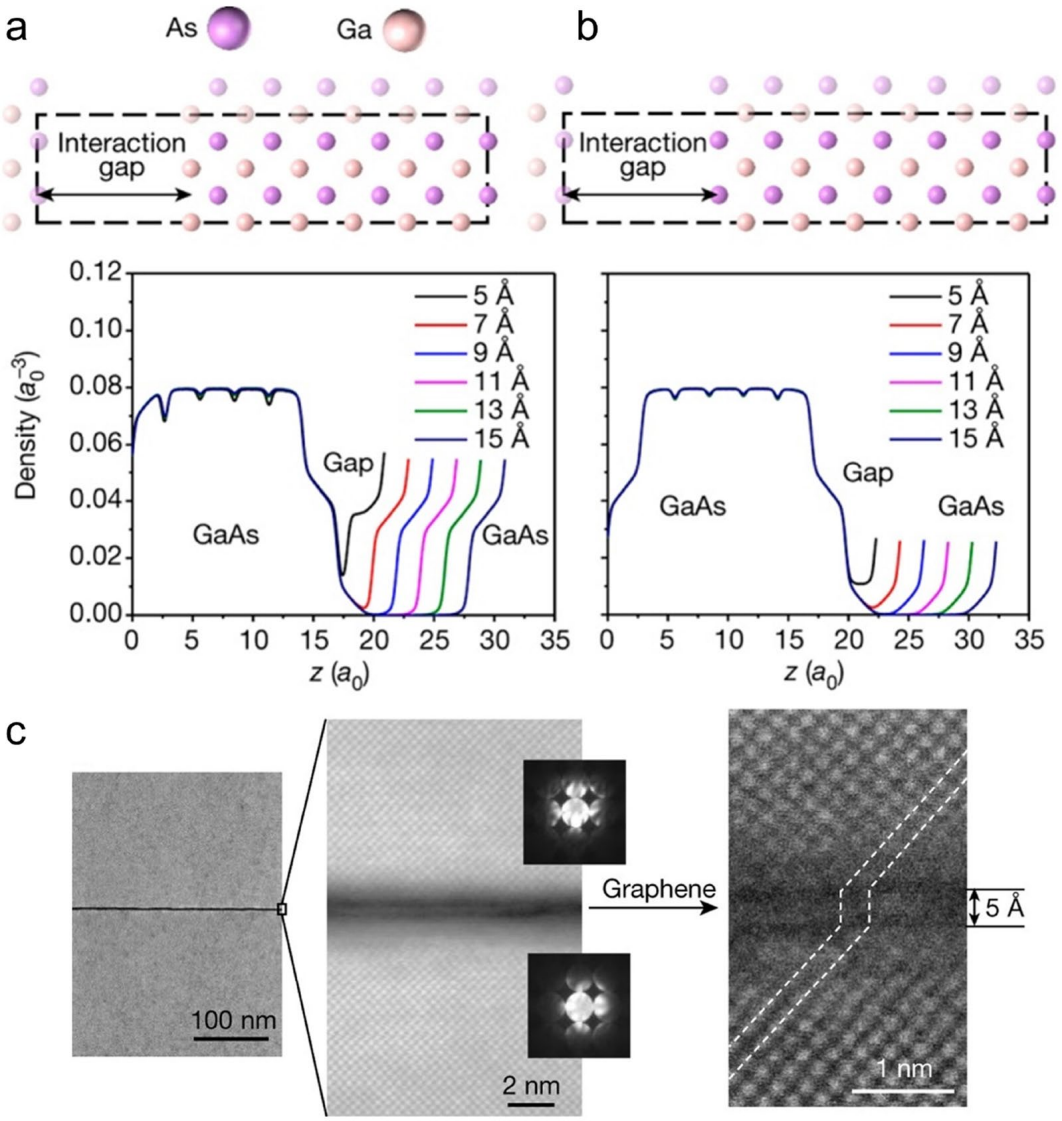
Substrate-epilayer remote interaction with different gaps created by different numbers of stacked graphene interlayers. Averaged electron density along separated slabs of GaAs for (**a**) As-Ga interaction and (**b**) As-As interaction. **c** High-resolution transmission electron microscopy (HRTEM) image of the GaAs/graphene/GaAs heterointerface [54]. Copyright from Springer Nature

Kim et al. first give a systematic explanation based on the concept of “lattice transparency” of graphene and name the modulation mechanism “remote epitaxy” for the crystals grown on the 2D-material-covered single-crystalline substrate [54]. To better illustrate that the crystallographic information of the substrate could be transmitted to the epilayer with the presence of the graphene interlayer, a cubic GaAs (001) crystal is selected to create a contrasting structure to the hexagonal graphene. Density functional theory (DFT) calculations are first performed to investigate the atomic interaction at the GaAs/GaAs heterointerface. It is found that the As-terminated GaAs (001) substrate could restrict the lattice of the GaAs epilayer through a substrate-epilayer gap up to 9 Å, which offers hope for maintaining the epitaxial relationship regardless of the graphene interlayer (Fig. 1a, b). Experiments are subsequently performed to validate their assumption. A single-crystalline GaAs epilayer is successfully obtained on the monolayer graphene-covered GaAs (001) substrate at a 5 Å substrate-epilayer gap as expected (Fig. 1c). While the ones grown on bi- and tri-layer graphene-covered GaAs (001) substrate are polycrystalline. This demonstrates that the atomic thin graphene is electrically penetrable, which allows the remote homoepitaxy to happen. Besides, the as-obtained epilayer could be easily exfoliated from the substrate due to the weak interaction at the arsenide/graphene interface, which offers the potential to stack and integrate multi-functional materials without the lattice matching limitation.

**Fig. 2:**
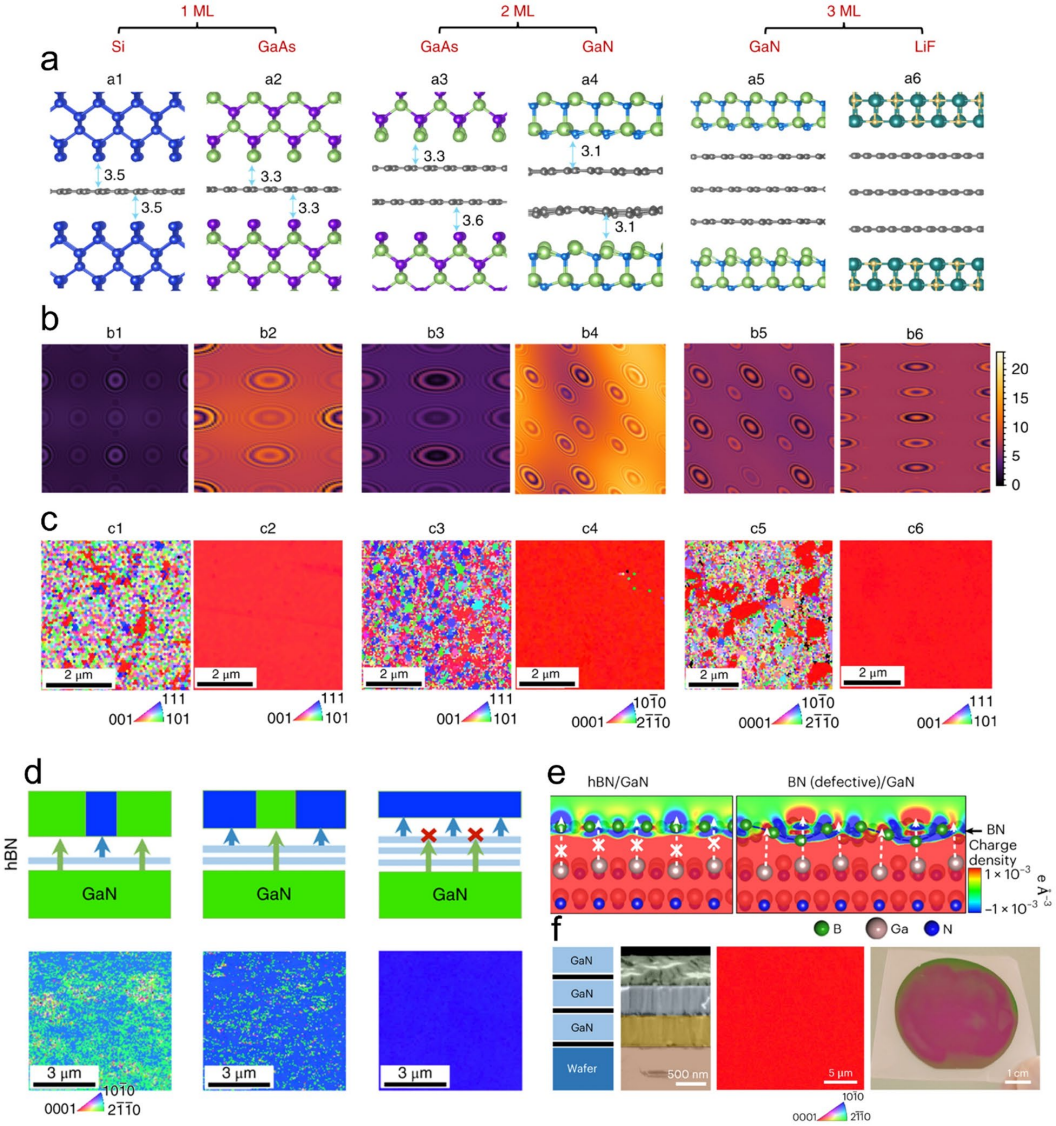
Penetration distance of the potential fluctuations from the Si, GaAs, GaN, and LiF substrates. **a** Atomic structures of Si, GaAs, GaN, and LiF on mono-, bi-, or tri-layer graphene-coated Si, GaAs, GaN, and LiF, respectively. The corresponding (**b**) maps of potential fluctuation on the same scale (0–25 meV) for cross-comparison and (**c**) EBSD of the released surfaces of each configuration. **d** Schematic and EBSD of the GaN epilayer grown on mono-, bi-, and tri-layer h-BN covered GaN substrate [55]. Copyright from Springer Nature. **e** Cross-sectional images of charge density distribution induced by GaN substrates with BN. **f** Schematic, cross-sectional false-colour SEM image, EBSD, and the exfoliated image of three stacks of GaN/BN grown on a GaN substrate [56]. Copyright from Springer Nature

The lattice transparency characteristic of graphene gives a first explanation of the lattice modulation mechanism in remote epitaxy: Graphene allows the potential field coming from the underlying crystalline substrate to penetrate it and modulate the subsequent epitaxial process. Motivated by this, Kong et al. begin to study how would the binding nature of the 2D materials and substrates affect the atomic interaction transmission process, aiming at providing general rules for the epilayer lattice modulation in remote epitaxy [55]. The effect of the bonding character of the crystalline substrate on the potential field penetrating ability is first studied. Remote homoepitaxy is implemented on monolayer graphene-covered GaN substrate and Si substrate, respectively. It turns out that the as-obtained GaN epilayer is single-crystalline on the GaN substrate, while it is poly-crystalline on the Si substrate. Considering that the insertion of the lattice transparent graphene would not affect the pattern of the substrate potential field, but changes the distance between the substrate and the growth front, the above-mentioned results should be attributed to the variation potential attenuation rate of different materials. For the pure covalent bonded Si, the potential of which follows the r^−6^ decay, whereas for the partial ionic bonded GaN, an r^−2^ decay is expected. As a result, after passing the substrate-graphene gap, the potential of the GaN substrate is still strong enough to guide the epitaxial process, but not for the Si (Fig. 2a–c). Besides, the binding nature of 2D material would affect its transparency to the substrate field. Taking GaN remote homoepitaxy as an example, a single-crystalline epilayer could be obtained with the presence of mono- and bi-layer graphene interlayer, but not for the thicker ones. While for the polar bonded h-BN, the monolayer h-BN would block the potential field of the GaN substrate and result in the failure of remote epitaxy (Fig. 2d). Recently, Kim et al. illustrate that the phase of the 2D material would also affect the substrate potential transmission process in remote epitaxy. DFT calculation results show that different from the case of single-crystalline h-BN, the defective BN interlayer allows the potential of the underlying GaN substrate to penetrate it and modulate the subsequent GaN epilayer growth (Fig. 2e). Experiments are subsequently exploited to explore the defective-BN-assisted GaN remote homoepitaxy. After growing a defective BN on the GaN substrate, a GaN epilayer is in situ grown in the MBE chamber. The as-obtained GaN epilayer turns out to be a single-crystalline one and had no epitaxial relationship with the BN, which verifies that the GaN epilayer is well-modulated by the underlying GaN substrate via remote epitaxy strategy. Furthermore, the in situ growth technique enables multi-stack growth and the epilayer could be precisely exfoliated at the heterointerface (Fig. 2f), which is favorable for producing high throughput single-crystalline semiconductor membranes [56].

These results indicate that the key to successfully implementing remote epitaxy is to ensure that the potential is strong enough to modulate the epilayer lattice after penetrating the 2D material and maintain the epitaxial relationship, which is closely related to both the binding nature of the substrate and the 2D material. For the substrates, the potential field penetrating ability is expected to be enhanced with increasing ionic bonding characteristics. When it comes to the remote epitaxy of LiF, there are more options available for the type and thickness of the 2D material buffer layer compared to Si. While for the 2D materials, the trend is the opposite and the nonpolar bonded graphene is more likely to lead a successful remote epitaxy. By selecting the 2D materials and the substrate pairs, the atomic interaction at the heterointerface in remote epitaxy could be modulated to adapt to various application scenarios. Following this instruction, many studies have achieved remote epitaxy on various material sets, including arsenide, nitrides, perovskite, and II-VI semiconductors [41, 46, 47, 51, 57–69]. The as-obtained epilayers perform well in the strain status, dislocation density, and heterointerface coupling strength, which is favorable for next-generation optoelectronics and electronics applications [70, 71].

**Fig. 3:**
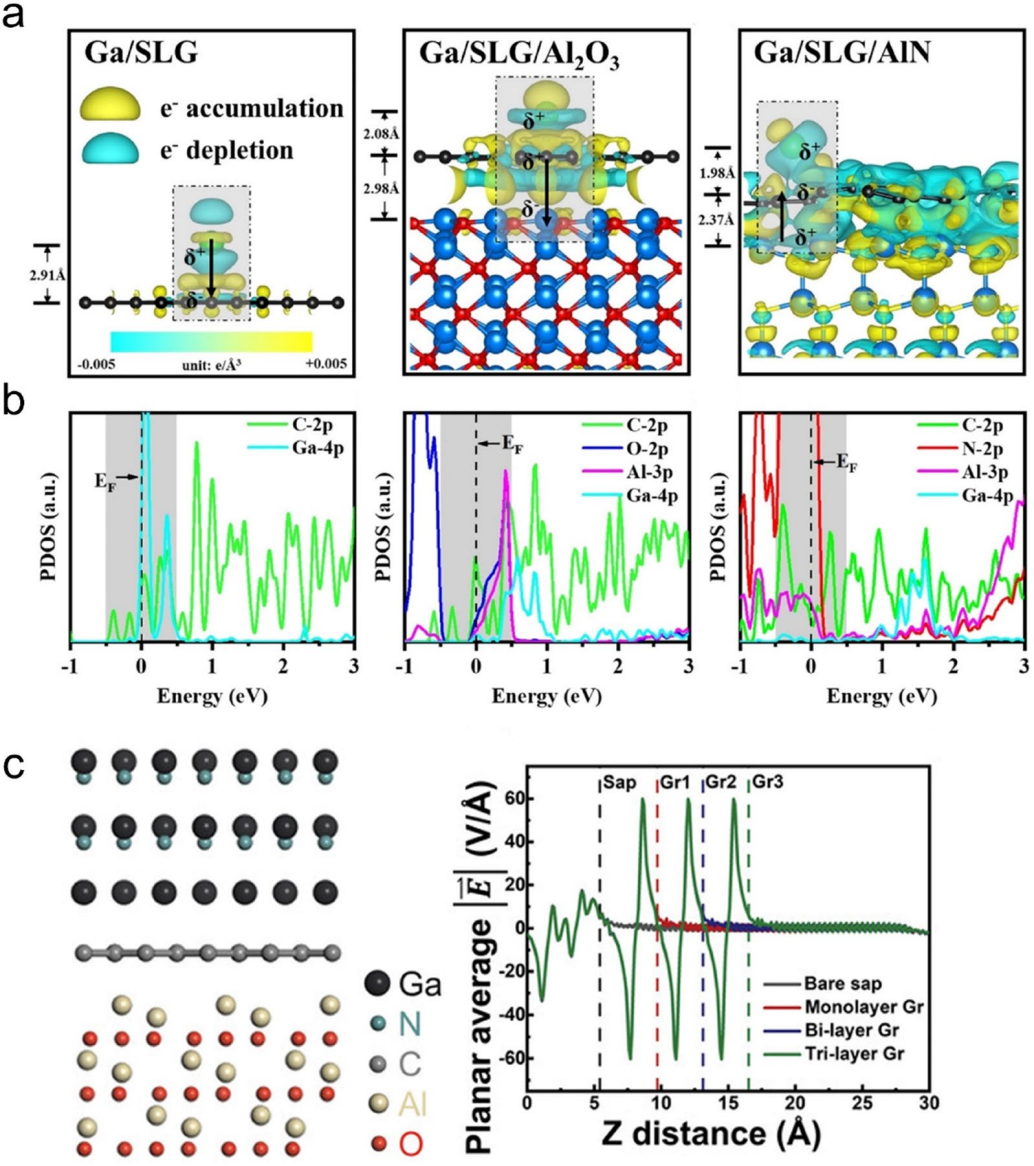
Lattice modulation mechanism in graphene-assisted nitrides remote epitaxy. **a** CDD of Ga/single-layer graphene, Ga/single-layer graphene/sapphire, and Ga/single-layer graphene/AlN at the H site. **b** PDOS of Ga/single-layer graphene, Ga/single-layer graphene/sapphire, and Ga/single-layer graphene/AlN at the H site [72]. **c** Atomic structure of sapphire/graphene/GaN interface and calculated planar average electric field of sapphire with various graphene layers. [73] Copyright from the American Chemical Society and Wiley‐VCH GmbH

After qualitatively revealing the strength of the interfacial interaction in remote epitaxy, studies are carried on to explore the essence of the remote interaction. After implementing GaN remote heteroepitaxy on monolayer graphene-covered sapphire substrate and AlN substrate, Qu et al. notice that the as-obtained GaN films are single-crystalline and the epitaxial relationship in remote heteroepitaxy remains the same with that in the heteroepitaxy [72]. This evidently indicates that the substrate still governs the epitaxial growth process, however, the separation of the substrate and epilayer makes the lattice-modulating pathway remain unclear in remote epitaxy. To answer this question, the nucleation behavior of GaN on the graphene-covered crystalline substrate is studied. Adsorption energy results indicate that to create a stable adsorbed initial layer, Ga adatoms are more suitable than the N adatoms and for the substrates, the one with more ionicity is preferred. To unveil the reason behind this, DFT calculation is subsequently exploited to study the atomic interaction at the GaN/graphene/crystalline substrate heterointerface. Charge density difference (CDD) results show that for the Ga/graphene heterointerface, electron depletion exists near the Ga side while electron accumulation near the graphene (Fig. 3a). Bader charge analysis illustrates that the binding nature of substrate would affect the amount of the transferred charge. Compared with sapphire, larger charge transfer is expected to happen at the GaN/graphene/AlN heterointerface due to the more ionic character of AlN. Partial density of states (PDOS) calculation is implemented to reveal the essence of the interfacial interaction in remote epitaxy. It is found that the orbital hybridization of Ga and AlN still exists regardless of the presence of the monolayer graphene (Fig. 3b). This long-range orbital hybridization effect reflects that in remote epitaxy, the epilayer and the substrate still could interact with each other through chemical interaction, which decides that it is the crystalline substrate, not the graphene, governs the remote epitaxial growth process.

This finding explains why the obtained remote epilayers usually have a satisfied crystalline orientation regardless of the rotation between graphene and the substrate, as well as the unchanged epitaxial relationship compared with that of homo- and hetero-epitaxy. Though this is inconsistent with the conventional lattice modulation wisdom, where the lattice guiding effect originating from the growth front is usually accepted, it could be explained by the lattice transparency behavior of graphene. In remote epitaxy, the presence of graphene could not block the lattice potential field coming from the crystalline substrate. This decides that though the epitaxial growth happens on the graphene surface, it is still led by the substrate lattice. However, considering that the insertion of graphene separates the epilayer and the substrate physically and the gap between them far beyond the covalent interaction distance, the formation of the covalent interaction seems unreasonable. To solve this problem, a deeper exploration of the role of graphene in remote epitaxy is needed.

Taking the nitrides/graphene/sapphire system as an example, Chen et al. focus on the behavior of graphene in the lattice modulation process of remote epitaxy. DFT calculation is performed to investigate the remote interaction between nitrides and sapphire [73]. It could be seen that the tendency of the average electric field remains unchanged after inserting the graphene layer, indicating that the presence of graphene would not affect the pattern of the potential field coming from the crystalline substrate. This verifies the existence of lattice transparency characteristic of graphene, which could be explained by the unique lattice structure of it. Graphene is a layered material without bond dipole moment neither in the in-plane nor in the out-of-plane direction, which allows the potential field of the crystalline substrate to penetrate it without changing the pattern. Besides, it is also found that after penetrating graphene, the crystalline potential field doesn’t attenuate inside the graphene as intended but continues to propagate with the same strength away from the graphene surface (Fig. 3c). This hints that the electric field is balanced inside the graphene, which should be attributed to the graphene pi electrons redistribution process. It also suggests that graphene might relay the propagation of potential field. As a result, the effective interaction distance between the substrate and the epilayer is increased and allows the long-range covalent interaction to happen in graphene-assisted remote epitaxy.

After these efforts, the lattice modulation strategy in remote epitaxy could be basically clarified. In remote epitaxy, though the insertion of 2D materials changes the growth front and enlarges the substrate-epilayer gap, the epilayer still inherits the lattice arrangement of the underlying crystalline substrate. This should be attributed to the novel properties possessed by 2D materials. First, the lattice transparency: It is believed that the 2D materials are transparent to the potential field of substrates, which decides the staking order of the subsequent epitaxial growth could be governed by the substrates [54, 55]. Second, the potential relay effect: Originating from the re-distribution of electrons, 2D materials, such as graphene, could maintain the potential field strength of substrates, which ensures the interaction between the epilayer and the substrate is still strong enough to guide the lattice arrangement of the epilayer [73]. Besides the lattice modulation mechanism in remote epitaxy, suggestions are also given for successfully implementing and bringing remote epitaxy to broader applications. First, select a suitable substrate. Remote epitaxy is not applicable to all the material sets. It is necessary to ensure that the strength of the substrate potential field is strong enough to guide the lattice of the epilayer, otherwise, one may obtain an epilayer with random orientation and unsatisfied quality. Considering the relationship between the potential attenuation rate and the binding nature of the crystalline substrate, the one with more ionicity is expected to promote a successful remote epitaxy to happen. Second, taking advantage of the lattice transparency characteristic and potential relay effect of the 2D material. The lattice transparency of 2D material allows the potential field coming from the crystalline substrate to penetrate it, meanwhile, the potential relay effect of the 2D materials ensures the penetrated potential could continue to propagate without attenuation. These together decide that the epilayer could be modulated by a potential field with the proper pattern and strength remotely. From this perspective, graphene is a more favorable choice for the 2D material buffer layer in remote epitaxy. Besides, to make these unique features of graphene into full play, the fabrication and transfer method should be designed carefully. Usually, a clean and sharp 2D material/substrate heterointerface is preferred for decreasing the potential disturbance and maximizing the remote electrostatic interaction [59, 74]. Thus, the usage of epitaxial graphene and dry transfer strategy is more likely to lead to a successful remote epitaxy.

### vdW epitaxy

Another member of the 2D-material-assisted epitaxy is the vdW epitaxy. Due to the lack of sufficient experiment results and a clear understanding of the modulation mechanism on the dangling-bond-free surface, it is used to roughly categorize the epitaxial growth happening on the 2D material surface, regardless of the crystalline information of the underlying substrate, as vdW epitaxy. However, it appears that this contradicts the initial concept of vdW epitaxy, which suggests that the dangling-bonds-free 2D/2D interface could remove the lattice constraint of conventional epitaxial growth. For the epitaxy on the 2D/3D complex substrate, though the dangling bonds on the growth front have been eliminated, the potential of the substrate, especially for the ones with more ionicity, could still govern the lattice arrangement of the epilayer [55]. The insertion of the 2D material doesn’t change the epitaxial relationship nor relieve the substrate restriction. From this perspective, it makes more sense to refer to certain instances of epitaxy, where the growth process is controlled by the potential field of the 2D material instead of the substrate below, as vdW epitaxy.

Abundant works are trying to break the substrate lattice constraint for bulk material growth using the vdW epitaxy strategy. Heilmann et al. use graphene as the buffer layer to implement GaN nanowires growth on Si (100) substrate [75]. The as-obtained GaN nanowires exhibit an ideal hexagonal shape without the visible structural defects, such as threading dislocation, inversion domain boundaries, or stacking faults, which indicate that the insertion of graphene changes the epitaxial relation and consequently breaks the lattice symmetry restriction between the epilayer and the substrate. Besides, compared with GaN grown on bare Si substrate, there are no observed misaligned microstructures and melt back etching for the GaN grown on the graphene-covered Si substrate, which demonstrates an additional advantage for the graphene layer, i.e., preventing the alloying between the Ga and Si during the MOCVD growth. Chung et al. successfully implement GaN-based microdisk LED growth on the Si/SiO_2_ substrate with a patterned graphene buffer layer [76]. The as-obtained GaN-based microdisk LED array exhibits visible blue light emission at room temperature and could be used for flexible device applications. Though there is no epitaxial relation between the substrate and GaN, the as-obtained GaN microdisk array is in a highly c-axis orientation. This indicates that an epitaxial relationship is constructed and graphene is capable of regulating the lattice of nitrides in the normal direction. While graphene couldn’t regulate the nitrides in-plane orientation and the GaN microdisks behave partially randomly orientated in the transverse direction.

These results indicate that vdW epitaxy is a powerful tool for implementing dissimilar materials integration, which is favorable for the next-generation semiconductor device and integrated circuit development. Besides, the possibility of growing crystals on any growth condition sustaining substrate via vdW epitaxy strategy is also verified, which may benefit the wide usage of large-scale and low-cost substrates in the semiconductor industry [77]. Along with the reported tremendous results, it is also found that the crystalline orientation of the structures fabricated via the vdW epitaxy strategy, especially in the in-plane direction, is not as satisfied as their counterpart, the remote epitaxy [78–82]. Early works of vdW epitaxy mainly focus on the nanostructure growth, which is discrete and small in size, thus, the crystalline orientation would not affect its performance dramatically [79, 83]. However, the performance of the membranes, which is regarded as fundamental for the device fabrication, is quite sensitive to the crystalline orientation. Studying and understanding the lattice modulation mechanism in vdW epitaxy is the key to broadening the application scenarios and fulfilling the advantages of it.

**Fig. 4:**
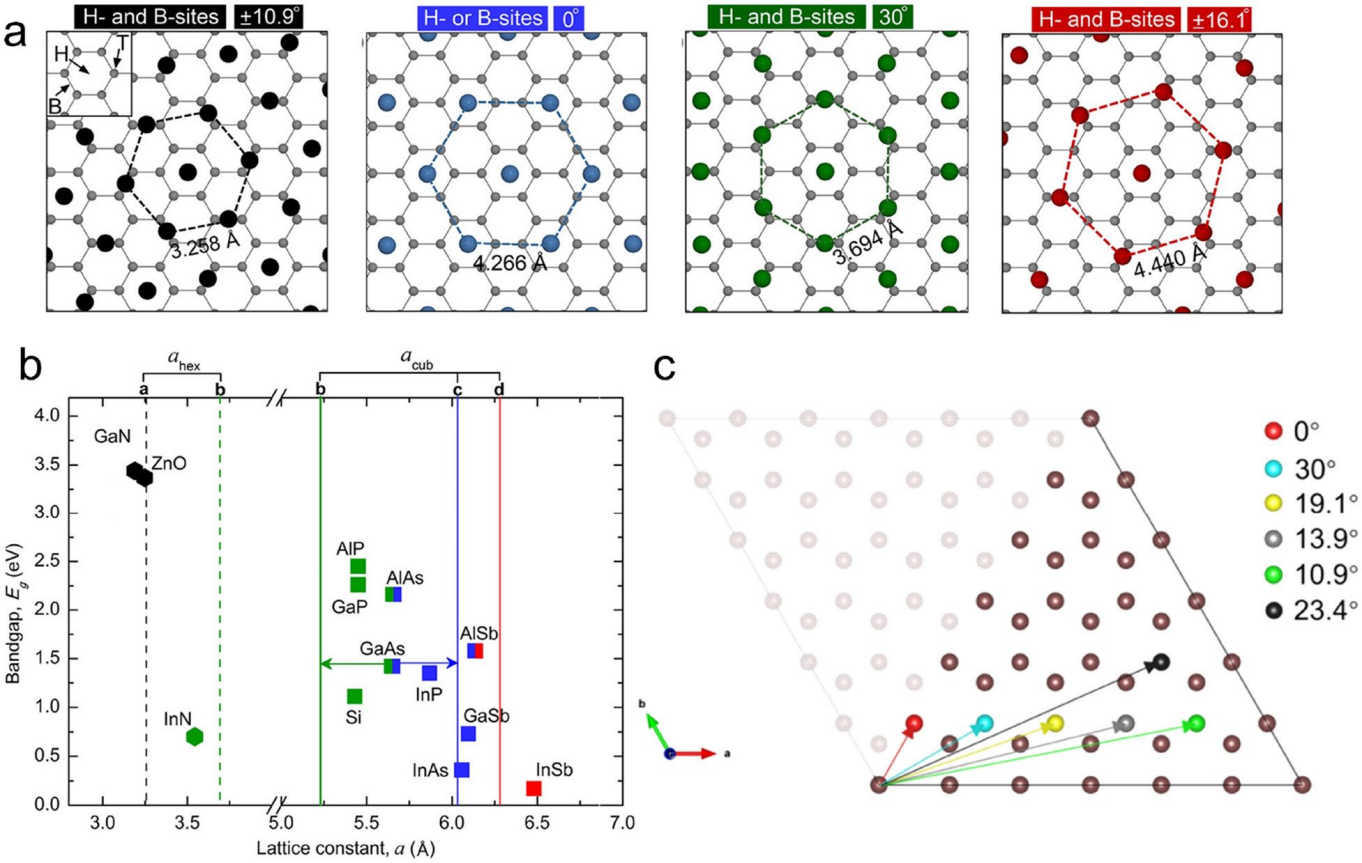
Possible adsorption sites and the corresponding epitaxial relationship for semiconductors on graphene. **a** Artificial lattice matched arrangement of the semiconductor atoms in the (111) plane of a cubic crystal [(0001) plane for hexagonal] when the atoms are placed above H- and B-sites and H- or B-sites. **b** Lattice constants for the lattice-matched atom arrangements. The square (■) and the hexagon (⬢) represent the cubic and hexagonal phases [84]. Copyright from American Chemical Society. **c** The possible lattice configurations according to the periodicity of the hexagonal lattice [85]. Copyright from American Association for the Advancement of Science

Munshi et al. give an explanation of the lattice modulation mechanism in vdW epitaxy based on the preferred adsorption sites analysis [84]. There are three high symmetry sites on the graphene surface, namely the H site (the center of the hexagonal carbon rings of graphene), the B site (the bridge between carbon atoms), and the T site (the top of a carbon atom). Because of the unfavorable adsorption of semiconductor adatoms on the T site, only the arrangements when the adatoms are placed on the (1) H site and B site and (2) H site or B site are taken into consideration. Correspondingly, several effective lattice constants are obtained and the rotation between the semiconductor and graphene could be calculated (Fig. 4a). Through comparing the lattice constant of the semiconductor and the as-obtained effective constant, one could infer the possible atomic arrangements at the heterointerface. For example, GaAs, the lattice constant of which is between two different atomic arrangements, i.e., adsorbing on the H site and B site with an effective lattice constant of 5.223 Å, 30° rotation and on the H site or B site with an effective lattice constant of 6.032 Å, 0° rotation. As a result, the GaAs grown on graphite tends to exhibit multi-in-plane orientation. While, ZnO and InAs, which is lattice matched to the atomic arrangement, namely adsorbing on the H site and B site with an effective lattice constant of 3.258 Å, ± 10.9° rotation, and on the H site or B site with an effective lattice constant of 6.032 Å, 0° rotation, respectively. Thus, it is possible to obtain ZnO and InAs single crystals on the graphite (Fig. 4b). To exclude the influence of the co-existing stacking order of graphene on the graphite surface, GaAs nanowires growth is exploited on the graphic substrate to verify the above-mentioned assumption. Through direct observation, it is found that two sets of nanowires, i.e., with 0° and 30° rotation, co-exist on the graphite surface, which is agree with the prediction. Besides, the amount of 0° nanowires is twice the number of the 30° ones, which could be explained by the relatively small lattice mismatch of the 0° configuration.

In vdW epitaxy, the interaction between the epilayer and the 2D material is expected to be a vdW one. Thus, at the initial epitaxial growth process, the demand of creating a covalent bond with a correct bond angle could be relieved and only the adsorptions sites need to be considered. Once the adsorption sites are decided, the subsequent lattice stacking process tends to follow the inherent sequence of the epilayer lattice. However, besides the lack of directivity, vdW interaction is also weak in strength. It is difficult to confine the adsorption of adatoms in vdW epitaxy to exactly predicted favorable adsorption sites, unlike in covalent epitaxy. Therefore, another explanation of the lattice modulation mechanism of vdW epitaxy, which is based on the lattice mismatch perspective, is proposed (Fig. 4c).

**Fig. 5:**
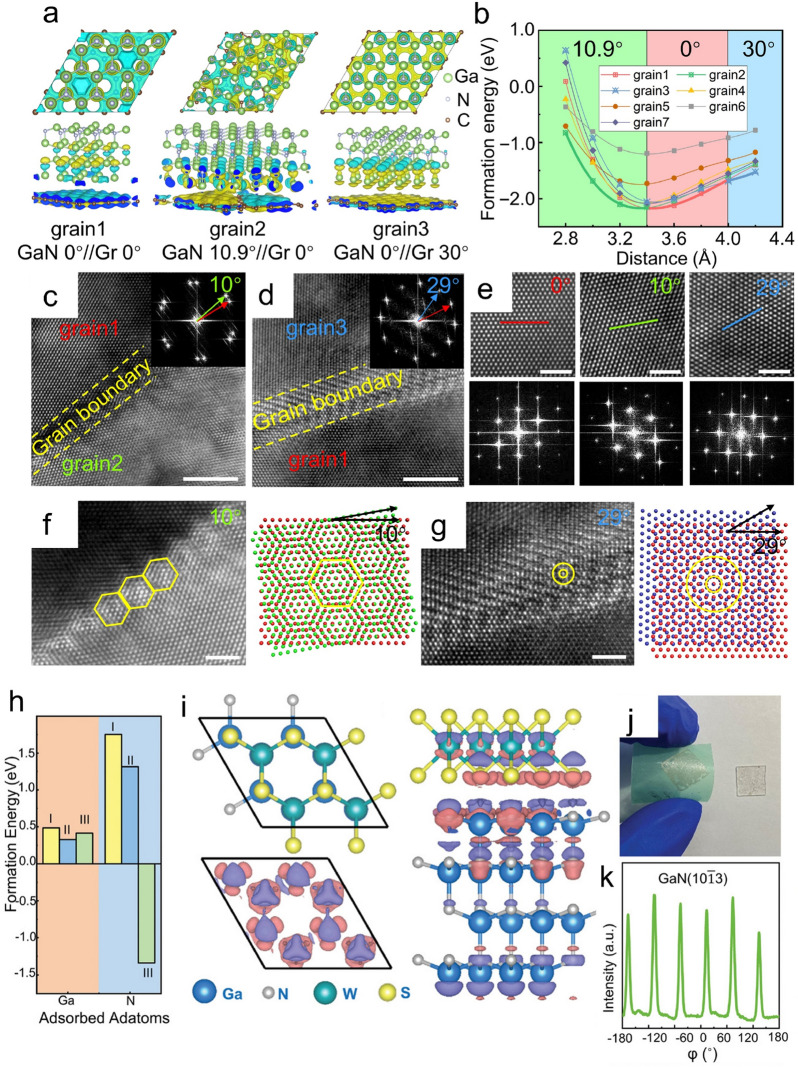
Lattice modulation mechanism in nitrides vdW epitaxy. **a** Calculated models and (**b**) Formation energy of different graphene/nitrides heterointerface configurations. **c**–**d** The grain boundary between grains with a specific rotation angle. **e** HRTEM and corresponding fast Fourier transform (FFT) images from each grain. **f**, **g** HRTEM images and structural representations of the Moiré patterns for the overlap of two GaN layers with relative rotations. Scale bars, 5 nm (c and d) and 2 nm (e to g) [85]. Copyright from American Association for the Advancement of Science. **h** The formation energy of three representative structures with N or Ga procedures on the WS_2_ surface. **i** Atomic model structures and CDD isosurfaces at the nitrides/WS_2_ heterointerface. **j** GaN epilayer after exfoliation. **k** XRD-ϕ scan of GaN (10 $\overline{1 }$ 3) direction. [94] Copyright from Wiley–VCH GmbH

Ren et al. study the GaN growth behavior on the graphene-covered amorphous quartz glass substrate [85]. According to the periodicity of the hexagonal lattice, there are 6 possible in-plane atomic arrangements with the rotation angle of 0°, 30°, 19.1°, 13.9°, 10.9°, and 23.4°. For each arrangement, the effective lattice constant of GaN and graphene could be calculated separately. Correspondingly, possible epitaxial relationships could be obtained as, GaN 0°//graphene 0°, GaN 10.9°//graphene 0°, GaN 0°//graphene 30°, GaN 19.1°//graphene 30°, GaN 13.9°//graphene 10.9°, GaN 30°//graphene 10.9°, and GaN 0°//graphene 19.1°. DFT calculations are performed to reveal the atomic behavior of the graphene-assisted nitrides vdW epitaxy (Fig. 5a, b). The binding energy results indicate that the interaction at the nitrides/graphene heterointerface exhibits a vdW feature. While, the difference in the formation energy between the above-mentioned configurations is relatively small, which hints that the in-plane orientation of nitrides is difficult to be regulated by graphene. To solve this problem, the nanorod-assisted vdW epitaxy strategy is applied. As a result, the as-obtained GaN epilayer is a nearly single-crystalline one with 3 preferred in-plane orientations (Fig. 5c–g). The subsequently deposited LED structure also shows good performance and could be exfoliated and transferred easily.

In contrast with graphene, 2D material TMDCs, such as WS_2_ and MoS_2_, are known to be lattice-matched with GaN [86–89]. Research is also carried out to study the TMDCs-assisted nitrides vdW epitaxy [90–92]. Gupta et al. try the nitrides growth on the Si/SiO_2_ substrate with the assistance of the mechanical exfoliated WS_2_ and MoS_2_ interlayer, respectively [93]. The as-obtained GaN islands are in regular hexagonal shape and with uniform in-plane orientation exhibiting a single-crystalline characteristic, which is quite different from the ones fabricated with the assistance of graphene [76, 78]. This implies that the lattice mismatch still plays an important role in the lattice modulation process of vdW epitaxy. Yin et al. successfully achieve single-crystalline GaN growth on the amorphous substrate via WS_2_-assisted vdW epitaxy and study the lattice modulation mechanism inside it. DFT calculations are performed to illustrate the role of WS_2_ in nitrides vdW epitaxy [94]. The adsorption of the adatoms on the WS_2_ surface is first investigated. The formation energy results illustrate that N adatoms adsorbing on the top of the W atoms is the only energetically favorable configuration (Fig. 5h). The interfacial binding energy is calculated to be 86 meV/ Å^2^, indicating a vdW binding feature. Then, CDD distribution and Bader analysis are exploited to study the WS_2_/nitrides heterointerface behavior. Different from the graphene/nitrides heterointerface, there is an sp^3^-like threefold symmetry with slight charge accumulation around the S atoms (Fig. 5i). The charge transfer is subsequently quantitively identified as that the N atom donates 0.075 e and the S atom donates 0.033 e per bond at the heterointerface, which is in agreement with the binding energy results. Based on these calculation results, the lattice modulation process in WS_2_-assisted nitrides vdW epitaxy could be described as: Guided by the potential field of the WS_2_, N adatoms adsorb on the top of the W atoms and interact with three S atoms via vdW force, which actually decides the epitaxial relationship between the nitrides and WS_2_. Meanwhile, the N atoms use the preserved 5.9 electrons to form covalent bonds with the Ga or Al atoms and sufficiently regulate the nitrides lattice. Nitrides growth on the WS_2_-covered amorphous substrate is implemented to verify the proposed mechanism. The as-obtained GaN film exhibits a single-crystalline characteristic with no residual strain and could be exfoliated easily, which confirms the remarkable lattice orientation guidance effect of the WS_2_ buffer layer and the weak vdW interaction at the heterointerface (Fig. 5j, k).

These results illustrate the advantage of vdW epitaxy in breaking the substrate restriction and flexible device fabrication [79, 95, 96]. Besides, the lattice modulation mechanism in vdW epitaxy is also revealed. As mentioned above, correctly placing the initial layer adatoms is the key step for the successful lattice modulation process in vdW epitaxy, which is closely related to the lattice mismatch between the 2D material and the epilayer. Take the nitrides vdW epitaxy as an example, there are multi-long-range lattice matching relationships co-existing at the graphene/nitrides heterointerface, which results in that graphene could not provide the only energetically favorable adsorption pattern for the nitrides, thus, fail to rightly arrange the initial layer of nitrides. Therefore, the nitrides grown via graphene-assisted vdW epitaxy tend to be polycrystalline. While, due to the nearly perfect lattice matching between nitrides and WS_2_, the initial nitrogen layer could unambiguously find the only proper adsorption sites and provide an ideal foundation for the subsequent nitrides lattice stacking process.

Based on this understanding, the key to sufficiently regulating the epilayer lattice in vdW epitaxy could be summarized as, choosing a lattice-matched, or more precisely, a geometry-matched 2D material as the buffer layer. A geometry-matched 2D material buffer layer could provide the only energetical favorable adsorption pattern for the initial layer of the epitaxial growth. Because there is no obvious orbital hybridization between the 2D material and the epilayer, the bond angle at the heterointerface could not be strictly restricted. The upcoming adatoms tend to bond with the initial layer and spontaneously form their own stable lattice sequence. For example, for a Ga pre-layer, with the upcoming N adatoms, a GaN structure is expected to be obtained; while with the As adatoms, a GaAs is expected. With a well-arranged initial layer, the nucleus on each adsorption site would grow and finally coalesce into a single-crystalline film.

Considering the epitaxial relationship is constructed between the epilayer and the 2D material, once a geometry-matched 2D material is found, with a 2D material transfer or direct growth process, one could construct a suitable growth front for the epilayer on any substrate. In this way, one could use vdW epitaxy to achieve target materials growth on arbitrary substrates. However, it should be noted that the influence of the substrate potential field should be excluded, which could be done by controlling the binding nature and thickness of the 2D material. Otherwise, it may disturb the 2D material potential and result in the failure of the vdW epitaxy. Besides, though the substrate only behaves like a mechanical support, its stability should also be considered. One should ensure the selected substrate could endure the growth condition, for example, the high temperature, oxidizing gas, or else the decomposition or diffusion of the substrate may result in the quality degradation of the epilayer.

### Quasi vdW epitaxy

Different from the remote epitaxy and vdW epitaxy, where the integrity of the 2D material needs to be preserved for sufficiently modulating the lattice of the epilayer, quasi vdW epitaxy usually uses an intentionally doped 2D material, which is found to bring novel impacts on the epitaxial growth process.

N-doped graphene is a widely used 2D material in the quasi vdW epitaxy [24, 97–103]. Chang et al. find that the N-doped graphene could change the growth kinetic and greatly boost the 2D growth mode for the AlN heteroepitaxy [104]. DFT calculation is performed to investigate the atom diffusion behavior and find that the insertion of graphene could sufficiently decrease the migration barrier and promote the lateral migration of Al adatoms on the growth surface when compared with that grown on the bare sapphire substrate. As a result, the AlN grown on N-doped-graphene-covered nanopatterned sapphire substrate could rapidly cover the concave cone and coalesce at a thickness of approximately 1 µm, which is favorable for solving the migration difficulty in AlN growth. Another problem in AlN film fabrication, the high residual stress, could also be alleviated by the N-doped graphene, namely the graphene-driving strain-pre-store engineering [105]. Different from the case on the bare sapphire substrate, in the nucleation stage, AlN nuclei on N-doped graphene tend to be higher in density and smaller in size. According to the DFT calculation results, due to the higher degree of freedom of the nanowire corner atoms, the corner bonds elongation in nanowires is larger than that in the infinite surface, which is profitable for the introducing tensile strain during the cluster coalescence process. Thus, it could be inferred that more tensile strain would be induced in AlN quasi vdW epitaxy when compared with the conventional heteroepitaxy. By adjusting the nucleation density of AlN on doped graphene, the generated tensile strain could be modulated in quasi vdW epitaxy. Using the large pre-stored tensile strain induced during the coalescence process to compensate for the lattice and thermal mismatch induced compressive strain, a strain-free and low dislocation density AlN film is successfully obtained on the N-doped-graphene-covered sapphire substrate.

**Fig. 6:**
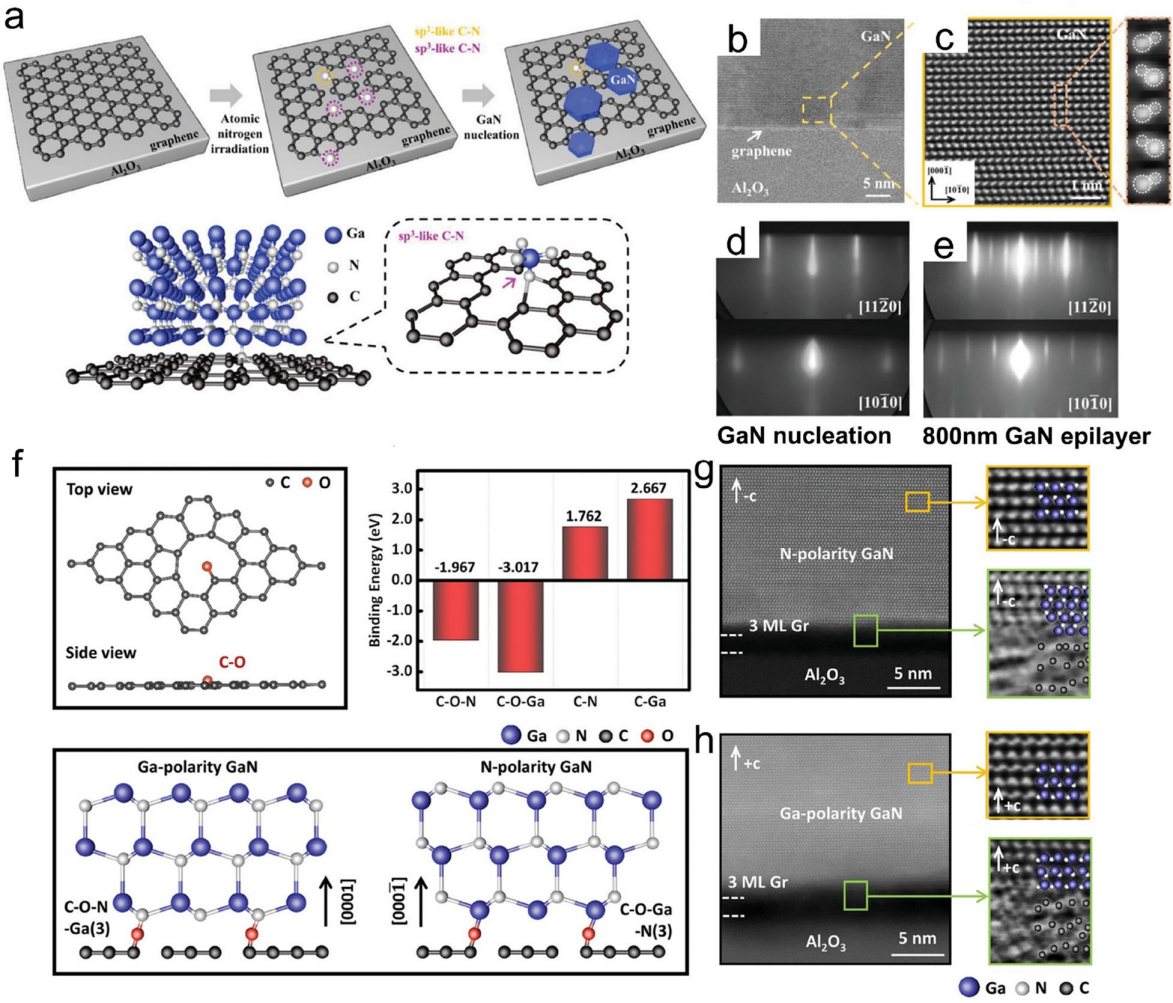
Lattice polarity modulation mechanism in nitrides quasi vdW epitaxy. **a** Schematic diagram of the nucleation growth of GaN on N-doped graphene covered sapphire substrate. **b** annular bright-field (ABF)-scanning transmission electron microscopy (STEM) image of GaN/graphene/sapphire interface. **c** integrated differential phase contrast (iDPC)-STEM image of GaN grown on N-doped graphene-covered sapphire substrate labeled by the frame in (**c**). In situ reflection high energy electron diffraction (RHEED) images of GaN films grown on N doped graphene covered sapphire substrate after (**d**) high-temperature GaN nucleation at 780 °C and (**e**) 800 nm-thick GaN epilayer cooling to 550 °C [106]. **f** Schematic diagram of the nucleation growth of GaN on O-doped graphene. **g** high-angle annular dark-field (HAADF)-STEM and iDPC -STEM images of the interface atomic structure of the N-polarity film. **h** HAADF-STEM and iDPC -STEM images of the interface atomic structure of the Ga-polarity film [107]. Copyright from Wiley–VCH GmbH

Besides, quasi vdW epitaxy also shows potential in lattice polarity modulation. Liu et al. find that on the N-doped graphene, it is more likely to obtain a N polar nitrides epilayer [106]. After nitrogen irradiation, C-N bonds are generated in graphene. The upcoming Ga atoms tend to bond with the sp^3^ C-N bonds to form C-N-Ga bonds. Subsequently, three N atoms will bond to Ga atoms and the C-N-Ga-N (3) structure is formed along the growth direction, which results in the as-obtained nitrides lattice of N polarity (Fig. 6a–e). While, with an O-doped graphene, one could manipulate the polarity of the nitrides [107]. The unsaturated C-O bonds on the graphene surface provide more options for the initial nitrides nucleation process, i.e., upcoming atoms bond with O atoms and form C-O-Ga bonds or C-O-N bonds, upcoming atoms replace the O atoms and form C-N bonds or C-Ga bonds. DFT calculations are performed to reveal the binding energy of the four possible atomic configurations. It is found that the C-O-Ga bond is the most stable configuration, which is expected to bond with three N atoms and form the C-O-Ga-N (3) structure of N polarity. The C-O-N configuration is also energetically favorable. Thus, it is also possible to obtain Ga polar GaN film on O-doped graphene via forming the C-O-N-Ga (3) structure. While for the C-N and C-Ga configurations, the binding energy of which is positive, indicating its instability (Fig. 6f). Experiment results further validate the above-mentioned nitrides lattice polarity modulation mechanism. Prioritizing Ga on the O-doped graphene, a N polarity GaN is obtained. In contrast, introducing N first will result in a GaN epilayer with Ga polarity (Fig. 6g, h).

These results illustrate that the usage of quasi vdW epitaxy could bring a new pathway to modulate the strain status and dislocation density in the epitaxial structure [108]. Besides, opportunities to artificially manipulate the lattice stacking sequence are provided by the quasi vdW epitaxy strategy, which would benefit the quality improvement of the epilayer and the design of new concept devices. However, the distorted 2D material is usually expected to introduce a disturbance to the substrate potential. How the complex potential coming from the distorted 2D material influences the subsequent epitaxial growth process is still an open question, which is worthy of exploration [109].

## Summary and outlook

Utilizing the unique and abundant properties of 2D materials, 2D-material-assisted epitaxy gives a chance to revolutionize the conventional epitaxial process. Improving our understanding of the lattice modulation mechanism in 2D-material-assisted epitaxy would have significant benefits for its practical application and future development.

Epitaxial growth on the 2D-material-covered crystalline substrate is usually regarded as the remote epitaxy. Ensuring the lattice guidance from the crystalline substrate is the key factor to successfully regulating the lattice of the epilayer in remote epitaxy, which is influenced by the substrate and the 2D material simultaneously. The substrate should have enough ionicity to preserve the strength of the potential after passing through the gap between the substrate and 2D material. This will allow for sufficient modulation of the epilayer lattice. Regarding 2D materials, those with lattice transparency and potential relay effects can maintain the pattern and strength of the potential field from the underlying crystalline substrate. This makes them ideal buffer layers in remote epitaxy. With the well-chosen substrate and 2D material companion, one could achieve transferrable and high-quality single crystal growth via the remote epitaxy strategy. Besides, remote epitaxy also shows potential in modulating the coupling strength at the 3D/2D/3D heterointerface, which offers a pathway to implement dissimilar material integration, even cross-dimensional ones.

Another 2D-material-assisted epitaxy strategy, the vdW epitaxy, is a promising candidate for achieving material growth on arbitrary substrates. By selecting the binding nature and the thickness of the 2D material, it is possible to block the potential field of the substrate and construct the epitaxial relationship between the epilayer and the 2D material. To make sure the lattice of the epilayer could be properly modulated by the inherent potential field of the 2D material, the lattice mismatch between the epilayer and the 2D material should be considered. To achieve a satisfactory crystalline orientation through the vdW strategy, it is crucial to have a lattice-matched 2D material buffer layer. This will ensure that the required adsorption pattern is energetically favorable and the first layer atoms could be correctly placed. By matching the lattice of the epilayer with the 2D material, combined with the 2D material transfer process, vdW epitaxy could break the long-standing substrate restriction in the heteroepitaxial growth and offer the hope for achieving the heterogeneous integration via the direct growth method.

Quasi vdW epitaxy is expected to happen on the artificially engineered 2D material surface. The modified sp^3^ and sp^2^ complex 2D material surface offers more possibilities for the 2D/3D interface construction process, which thus brings opportunities to design and control the characteristics of the as-obtained epilayer, such as the strain status and lattice polarity, and subsequently offers freedom for the device fabrication.

In summary, 2D-material-assisted epitaxy provides a fertile playground for creating hybrid systems with unique functionalities and excellent performance. Based on these valuable experimental and theoretical achievements of 2D-material-assisted epitaxy, one could better understand and properly utilize the 2D-material-assisted epitaxy strategy in different application scenarios, which may give inspiration for the design of new type devices and next-generation integrated systems.

## Data Availability

The data used in this work are available from the authors on reasonable request.
